# Therapeutic activity of green synthesized selenium nanoparticles from turmeric against cisplatin-induced oxido-inflammatory stress and cell death in mice kidney

**DOI:** 10.1042/BSR20231130

**Published:** 2023-11-10

**Authors:** Barakat M. ALRashdi, Roaya A. Mohamed, Amal H. Mohamed, Feryal A. Samoul, Mazen I. Mohamed, Mohsen M. Moussa, Saad M. Alrashidi, Bassel Dawod, Ola A. Habotta, Ahmed E. Abdel Moneim, Shimaa S. Ramadan

**Affiliations:** 1 Department of Biology, College of Science, Jouf University, Sakaka 72388, Saudi Arabia; 2 Department of Chemistry, Faculty of Science, Helwan University, Cairo, Egypt; 3 Consultant Radiation Oncology, Comprehensive Cancer Centre, King Fahad Medical City and College of medicine, Alfaisal University, Riyadh, Saudi Arabia; 4 McMaster Children’s Hospital, Faculty of Health Sciences, Hamilton, Ontario, Canada; 5 Department of Biology, College of Science, Jouf University, Sakaka, Al-Jouf, Saudi Arabia; 6 Department of Zoology, Faculty of Science, Fayoum University, Fayoum, Egypt; 7 Department of Forensic Medicine and Toxicology, Faculty of Veterinary Medicine, Mansoura University, Mansoura, Egypt; 8 Zoology and Entomology Department, Faculty of Science, Helwan University, Cairo, Egypt

**Keywords:** cisplatin, inflammation, nephrotoxicity, oxidative stress, selenium nanoparticles, turmeric extract

## Abstract

Cisplatin (CDDP) is a commonly prescribed chemotherapeutic agent; however, its associated nephrotoxicity limits its clinical efficacy and sometimes requires discontinuation of its use. The existing study was designed to explore the reno-therapeutic efficacy of turmeric (Tur) alone or conjugated with selenium nanoparticles (Tur-SeNPs) against CDDP-mediated renal impairment in mice and the mechanisms underlying this effect. Mice were orally treated with Tur extract (200 mg/kg) or Tur-SeNPs (0.5 mg/kg) for 7 days after administration of a single dose of CDDP (5 mg/kg, i.p.). N-acetyl cysteine NAC (100 mg/kg) was used as a standard antioxidant compound. The results revealed that Tur-SeNPs counteracted CDDP-mediated serious renal effects in treated mice. Compared with the controls, Tur or Tur-SeNPs therapy remarkably decreased the kidney index along with the serum levels of urea, creatinine, Kim-1, and NGAL of the CDDP-injected mice. Furthermore, Tur-SeNPs ameliorated the renal oxidant status of CDDP group demonstrated by decreased MDA and NO levels along with elevated levels of SOD, CAT, GPx, GR, GSH, and gene expression levels of HO-1. Noteworthy, lessening of renal inflammation was exerted by Tur-SeNPs via lessening of IL-6 and TNF-α besides down-regulation of NF-κB gene expression in mouse kidneys. Tur-SeNPs treatment also restored the renal histological features attained by CDDP challenge and hindered renal apoptosis through decreasing the Bax levels and increasing Bcl-2 levels. Altogether, these outcomes suggest that the administration of Tur conjugated with SeNPs is effective neoadjuvant chemotherapy to guard against the renal adverse effects that are associated with CDDP therapy.

## Introduction

Cisplatin (CDDP), a platinum derivative, is a widely used chemotherapeutic agent to treat various types of tumors such as ovary, bladder, testis, breast, and lung cancers [1,2]. CDDP exerts its action by forming cross-links in the purine bases, resulting in disturbance in DNA and RNA replication and impairment in cellular division [3,4]. Despite its effective anti-tumor activity, its clinical use is restricted due to serious toxic effects, of which renal damage is the main sequel [4,5]. In addition to its action on tumor cells, CDDP also targets normal somatic cells, particularly in kidneys, as being the main route of CDDP excretion and accumulation [6]. According to statistics, approximately 20–30% of CDDP-treated patients have developed severe renal dysfunction. Thus, effective therapeutic strategies are urgently needed to combat or prevent CDDP-induced renal injury.

The mechanisms of CDDP-induced renal impairment are complicated. Oxidative and inflammatory stresses besides cell apoptosis and necrosis are considered the primary hallmarks of CDDP-nephrotoxicity [4,5]. Reactive oxygen species (ROS) are generated under CDDP therapy resulting in damage to kidney tubular epithelial cells and loss of renal function [2]. CDDP was reported to induce a marked reduction in nuclear-factor-erythroid-2-related factor 2 (Nrf2), which is a vital regulator for other antioxidant enzymes such as hemeoxygenase-1 (HO-1) [7]. Moreover, CDDP was accused of producing excess pro-inflammatory cytokines and triggering nuclear factor kappa-B (NF-κB) signaling that exacerbates further tissue damage [8]. In addition, CDDP-treated renal epithelial cells displayed notable activation of caspases and mitochondrial translocation of Bax with subsequent tubular cell death [6–8]. Hence, it is a reasonable therapeutic approach to protect against the toxic effects of CDDP via inhibition of oxidative, inflammatory, and apoptotic signaling pathways.

Turmeric (*Curcuma longa L*.), a member of Zingiberaceae family, has been used since ancient times as traditional food, spices, and phytotherapy for the treatment of anorexia, infectious diseases, gastric, hepatic and renal disorders [9]. Moreover, it has a myriad of pharmacological activities, such as antimicrobial, antidepressant, anticancer, anti-inflammatory. and wound healing effects [10]. The ethanolic turmeric extract exerted significant neuroprotection against trimethyltin-induced oxidative stress in rat brain by increasing the activities of antioxidant enzymes [11]. Similarly, turmeric extract protected the liver from oxidative stress mediated by carbon tetrachloride [12], acute ethanol [13], and methotrexate [14] in experimental animals. Russo and collaborators found that oral administration of turmeric resulted in a significant decrease in urinary inflammatory markers and renal histopathological alterations caused by doxorubicin in rats [15]. Additionally, curcumin, an active ingredient of the turmeric extract, has been reported to ameliorate against nephrotoxicity caused by zinc oxide nanoparticles [16], cadmium [17], arsenic [18], and cisplatin [19]. Recently, Alvarenga et al. [20] reported that treatment with curcumin for 12 weeks resulted in significant decline in the plasma levels of tumor necrosis factor-α (TNF-α) of chronic kidney disease patients.

Unfortunately, curcumin bioavailability is low and its water solubility is very poor. In comparison to free curcumin, nanostructured curcumin has a higher systemic bioavailability in the plasma and tissues, according to Zou et al. [21]. Furthermore, compared to therapy with native curcumin, nanocurcumin has a 60-fold longer biological half-life and enhances *in vivo* absorption and dispersion [22]. Moreover, several formulations of nanoparticle-based curcumin were non-toxic in acute toxicity studies at doses equivalent to 2 g curcumin/kg of body weight. In this regard, the daily administration of a curcuminoid-essential oil complex at a dose of 1 g/kg body weight revealed no toxicity after 90 days [23] and Jantawong et al. [24] found that the oral median lethal dose (LD_50_) values of curcumin-loaded nanocomplexes estimated using Lorke’s method were 8.9 and 16.8 g/kg bw (equivalent to 2.5 and 4.5 g/kg bw of curcumin) for mice and hamsters, respectively.

Nanosized selenium particles (SeNPs) have attained much interest owing to their high bioavailability, lower toxic effects, and pronounced biological activities [25,26]. SeNPs is highly safe in Wistar rats up to 5000 mg/kg and in mice up to 2000 mg/kg according to Khubulava et al. [27] and Lesnichaya et al. [28], respectively. Nano-selenium supplementation mitigated diabetic nephropathy induced oxidative stress and restored renal structure and function in pregnant rats [29]. Likewise, SeNPs reversed vancomycin-induced renal toxicity via their antioxidant, anti-inflammatory, anti-apoptotic, and mitochondrial regulatory effects [30]. Notably, previous studies have reported that eco-friendly synthesized SeNPs using herbal extracts elicited noteworthy antioxidative and anti-inflammatory activities owing to the synergism between selenium and phytochemicals [31,32]. For example, SeNPs biosynthesized from the endophytic fungus *Fusarium oxysporum* displayed notable antioxidant and anti-apoptotic activities against doxorubicin-induced damage in liver, kidney and heart tissues of Swiss mice [33]. Remarkable nephroprotective efficiency was observed for green synthetized SeNPs against melamine [34] and acetaminophen [35] toxicities in experimental rats.

Based on the above, this study was conducted to inspect the possible mitigating potency of Tur synthesized SeNPs against CDDP undesirable renal effects. The mechanisms underlying this effect involving oxidative stress, inflammation, and apoptosis were also elucidated in the kidney of treated mice.

## Materials and methods

### Reagents

CDDP, Na_2_SeO_3_, and N-acetyl cysteine (NAC) were of analytical grade and were obtained from Sigma Chemical Co. (St. Louis, U.S.A.).

### Preparation of turmeric extract

Turmeric aqueous extraction was prepared by boiling 100 g of Tur in 1 L distilled water for 15 min. Then, the solution was allowed to cool for 20 min. After cooling, the prepared solution was filtrated and the filtrate was then used to prepare the required doses [36].

### Preparation and characterization of Tur- SeNPs

#### Preparation of SeNPs

Approximately 5 ml of turmeric aqueous extract (0.1 mM/ml) was mixed with Na_2_SeO_3_ (0.1 mM/ml) at 18°C and stirred. Two drops of vitamin C (0.1 mM) were added to enhance the formation of SeNPs. The change from blue to deep red implies that SeNPs were successfully formed. A Zetasizer and potential were used to determine the average size and charge of the synthesized nanoparticles, which revealed that the average diameter of the particles was 15.3 nm and a zeta potential of −12.2 mV (data not shown). In addition, Fourier Transform Infra Red spectroscopy (FTIR; PerkinElmer, U.S.A.) was performed to determine the main functional groups in the formed particles, which revealed the conjugation of Se and C atoms of Tur evidenced by the presence of C-X stretching bands at 435.62, 427.51, and 407.49 cm^−1^ (data not shown).

### Animal and ethical statement

Male Swiss albino mice of body weight ranging from 20 to 26 g and 8-week-olds were obtained from VACSERA animal facility (Cairo, Egypt). The mice underwent a one-week acclimatization period in a conventional laboratory setting with 12 h light/dark cycles, a temperature of 25°C, and unrestricted access to water and pelleted rodent food. The Institutional Animal Care and Use Committee (IACUC) of Helwan University (approval number HU2021/Z/AES0921-02) approved all experimental protocols and techniques. All animal experiments took place at Zoology and Entomology Department, Faculty of Science, Helwan University (Cairo, Egypt).

#### Treatment protocol

The mice were allocated into five groups (7 mice/group) as follows:

The first group (control): this group was orally given normal saline (0.9% NaCl solution) for seven days.
The second group (CDDP group):this group received i.p. injection of 5 mg/kg body weight (b.wt.) of CDDP, as previously reported by Abdel Moneim [37].
The third group (CDDP + Tur): received i.p. injection of CDDP at 5 mg/kg b.wt. and an hour later, mice were orally administered with turmeric extract at a dose of 200 mg/kg b.wt [36].
The fourth group (CDDP + Tur-SeNPs group): The animals were intraperitoneally injected with 5 mg/kg b.wt. CDDP and then given 0.5 mg/kg b.wt. of turmeric extract loaded SeNPs orally an hour later.
The fifth group (CDDP + NAC): animals received an i.p. injection of CDDP at 5 mg/kg b.wt., followed by an oral 100 mg/kg bw of N-acetylcysteine (NAC) as a reference drug after one hour [38].

The administration of these corresponding doses took place every day for 7 days. Within 24 h of the last treatment, mice were euthanized following an intraperitoneal injection of ketamine/xylazine (100 and 10 mg/kg b.wt., respectively). Blood was immediately drawn, stored at 37°C for 24 min, centrifuged for 15 min at 3000 rpm, and the serum was kept at −20°C. The left kidney from the animals was used for histological analysis after the kidneys were removed. The half of right one underwent homogenization with 10 mM PBS, centrifugation for 12 min at 3600 rpm, and storage of the supernatant at −80°C for biochemical examinations. Whereas, the remaining half was stored at --80°C for molecular measurements.

### Assessment of relative kidney weight

The relative kidney weight was calculated according to the following equation [26]:

Relative kidney weight = (Left kidney weight/Body weight)×100

### Kidney function test

The levels of urea and creatinine were measured in the serum based on the manufacturer’s information using kits obtained from Randox/Laboratory, Crumlin, UK.

### Assessment of kidney injury molecule-1 (Kim-1) and neutrophil gelatinase-associated lipocalin (NGAL)

ELISA kits were utilized for analysis of the serum levels of Kim-1 (R&D Systems, Catalogue Number: AF3689), and NGAL (MyBioSource, Catalogue Number: MBS260195) as mentioned in the manufacturers’ protocols.

### Analysis of renal oxidative stress markers

Lipid peroxidation (LPO) in the renal samples expressed in MDA concentrations was measured as stated by the method of Yagi et al. [39], while nitric oxide (NO) concentrations were measured based on the methodology of Green et al. [40]. Furthermore, glutathione (GSH) levels also were measured according to the protocol of Akerboom and Sies [41].

### Assessment of renal antioxidant enzymatic activity

The enzymatic activities of catalase (CAT), superoxide dismutase (SOD), glutathione reductase (GR), and glutathione peroxidase (GPx) in renal samples were evaluated using Aebi [42], Misra and Fridovich [43], Pinto et al. [44], and Tappel [45], correspondingly.

### Analysis of renal pro-inflammatory cytokines

The renal inflammatory response to CDDP injection and different treatments was evaluated by measurement of TNF-α (Catalogue Number: NBP1-92681) and interleukin-6 (IL-6; Catalogue Number: NBP1-92697) using ELISA kits obtained from Novus Biologicals (Centennial, CO, U.S.A.).

### RNA extraction, cDNA synthesis, and quantitative RT-PCR

Total RNA was extracted from renal tissue by TRIzol reagent, followed by cDNA synthesis using RevertAid H Minus Reverse Transcriptase (Fermentas, Thermo Fisher Scientific Inc., Canada) as mentioned in the manufacturer’s protocol. qRT-PCR was employed using the QuantiFast SYBR Green RT-PCR kit (Qiagen, Hilden, Germany). All reactions were conducted in duplicate using the ViiA7 System (Thermo Fisher Scientific, CA, U.S.A.). The PCR cycling conditions included initial denaturation at 95°C for 12 min, followed by 40 cycles of denaturation at 94°C for 60 s and annealing at 58°C for 60 s, extension at 72°C for 90 s, then holding for a final extension at 72°C for 10 min. The relative gene expression was determined between the different groups using the ^ΔΔ^Ct method. The primer sequences (Jena Bioscience [Jena, Germany]) for estimation of NF-κB and HO-1 gene expression levels were adjusted to GAPDH (housekeeping gene). Table 1 lists the primer sequences.

**Table 1:** Primers sequences

Name of the gene	Forward primer (5′-3′)	Reverse primer (5′-3′)
**NF-kB**	GCGTACACATTCTGGGGAGT	CCGAAGCAGGAGCTATCAAC
**HO-1**	TTAAGCTGGTGATGGCCTCC	GTGGGGCATAGACTGGGTTC
**GAPDH**	AGTGCCAGCCTCGTCTCATA	GATGGTGATGGGTTTCCCGT

### Estimation of renal apoptotic markers

The levels of apoptosis-related components in renal tissues, BCL2-associated X protein **(**Bax), and B-cell lymphoma 2 (Bcl-2), were measured using mouse ELISA kits (My BioSource, SD, U.S.A.) based on the manufacturer’s instructions.

### Renal histopathological examination

The left kidneys were fixed in 10% neutral buffered formalin for 24 h. Next, tissue samples were dehydrated, embedded in paraffin, and finally sectioned at 5 μm thick sections. Renal specimens were stained with H&E and examined under a Nikon Eclipse E200-LED (Tokyo, Japan) microscope at 400× magnification.

### Statistical analysis

For the collected data, statistical analysis was carried out using SPSS version 23. Resulted data were subjected to one-way analysis of variance (ANOVA) followed by Duncan’s post-hoc multiple tests to determine the differences between groups. The obtained results were displayed as mean ± standard deviation (SD). The statistical significance was determined when *P*-values were less than 0.05 (*P*<0.05).

## Results

### Effect of Tur and Tur-SeNPs on kidney index and function markers in CDDP-injected mice

**Figure 1:**
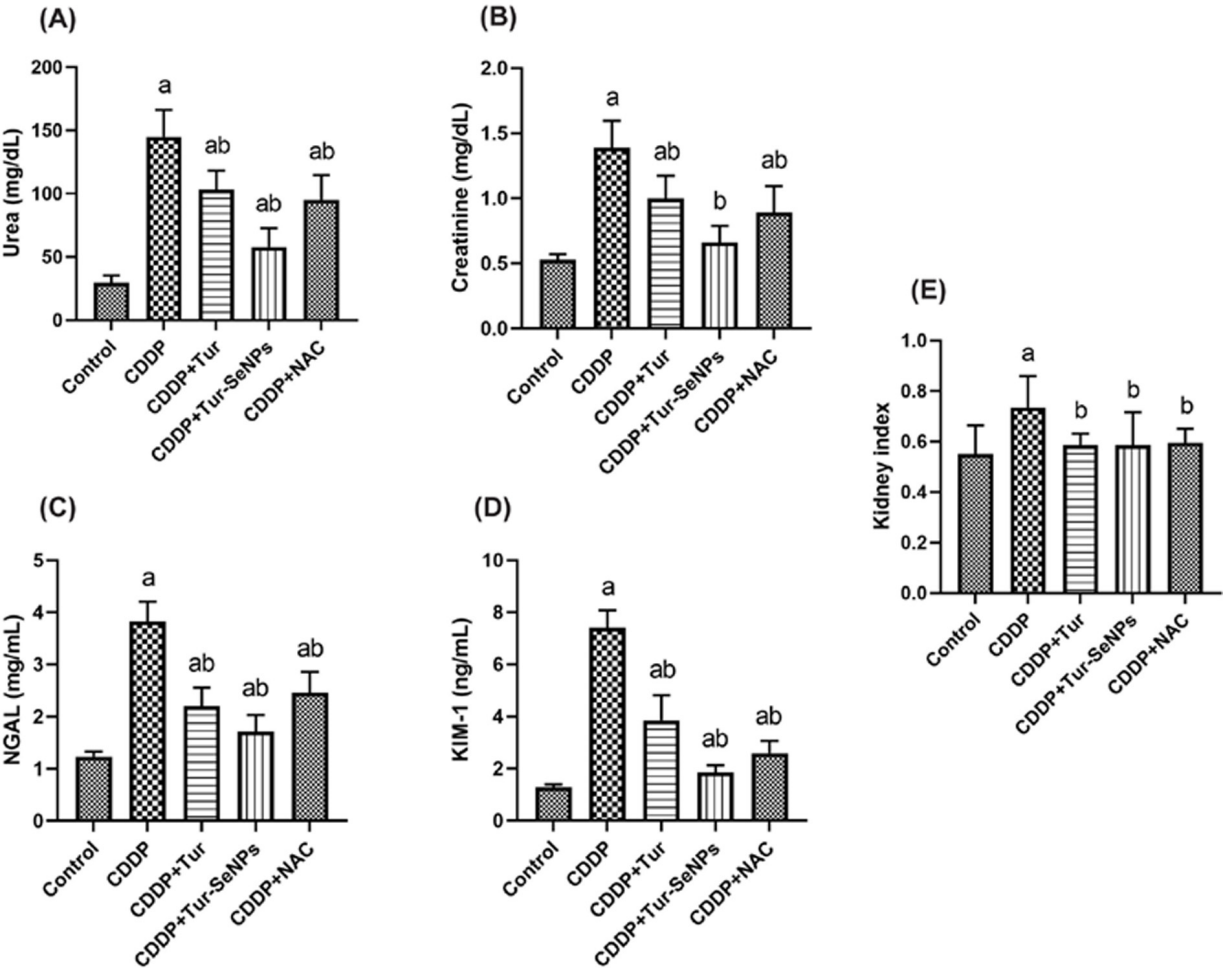
Effects of orally administered turmeric (Tur), turmeric-loaded selenium nanoparticles (Tur-SeNPs), or N-acetyl cysteine (NAC) on renal function markers [(A) urea, (B) creatinine, (C) Kim-1, and (D) NGAL] and (E) kidney index in cisplatin (CDDP)-injected mice Data are expressed as mean ± SD (*n*=7). a and b denote significant differences (*P*<0.05) compared with the untreated control and CDDP-treated groups, respectively.

The nephroprotective ability of Tur or Tur-SeNPs against CDDP-caused changes in kidney index and function indices is shown in Figure 1. CDDP injection induced a substantial increase (*P*<0.05) in kidney index related to the control group. Noteworthy, treatment with Tur, Tur-SeNPs, or NAC restored (*P*<0.05) the kidney near to the control value. Furthermore, CDDP administration caused dramatic increases (*P*<0.05) in serum creatinine and urea compared with the sham group. Meanwhile, administration of Tur, Tur-SeNPs, or NAC to CDDP-injected mice notably (*P*<0.05) reversed these markers compared to the CDDP group. Notably, Tur-SeNPs therapy was able to lessen the serum creatinine value in CDDP-injected mice close to the control value, which may explain their high nephroprotective efficiency.

Moreover, we measured the levels of Kim-1 and NGAL as being indicators for renal injury and toxicity in respect to the routinely used serum urea and creatinine. When compared with the controls, substantial rises (*P*<0.05) were observed in the levels of Kim-1 and NGAL following CDDP injection. However, treatment with Tur, Tur-SeNPs, or NAC counteracted these changes, where Kim-1 and NGAL were reduced (*P*<0.05) in comparison to the CDDP-treated group.

### Effect of Tur and Tur-SeNPs on renal oxidant/antioxidant status of CDDP-injected mice

**Figure 2:**
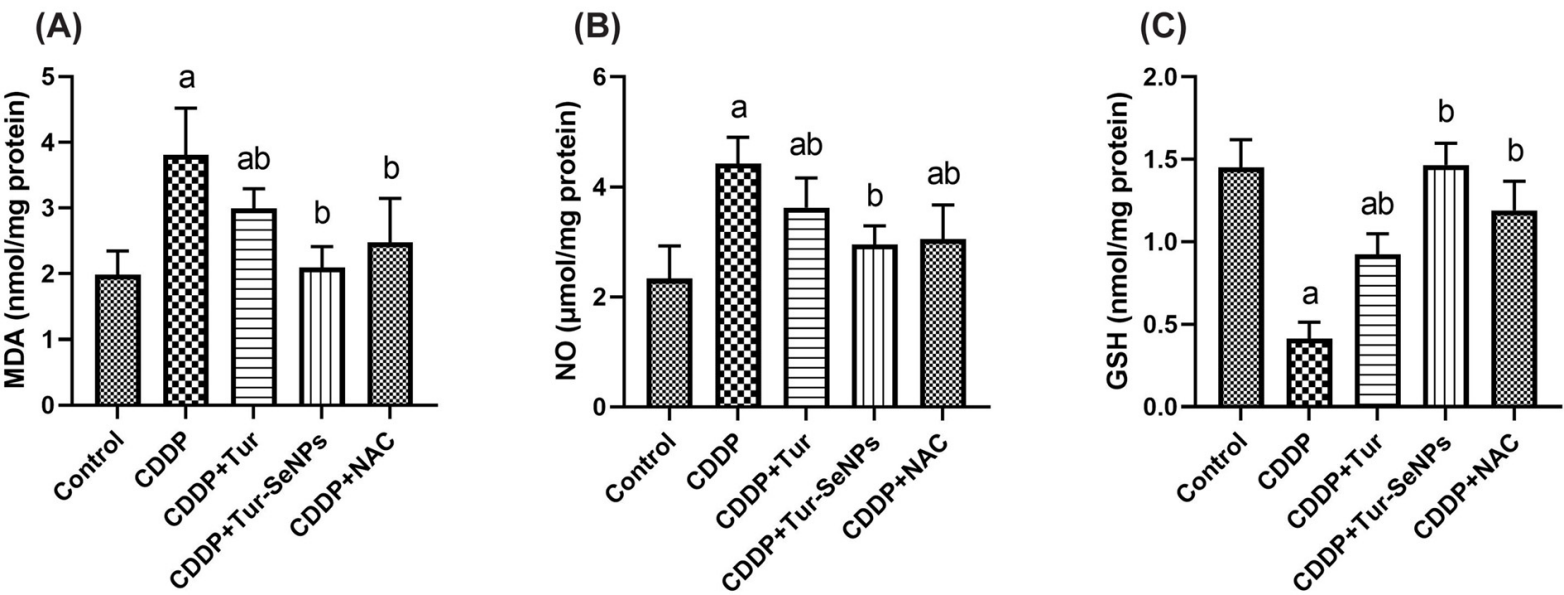
Effects of orally administered turmeric (Tur), turmeric-loaded selenium nanoparticles (Tur-SeNPs), or N-acetyl cysteine (NAC) on the levels of non-enzymatic oxidative stress markers [(A) MDA, (B) NO, and (C) GSH] in the kidney of cisplatin (CDDP)-injected mice Data are expressed as mean ± SD (*n*=7). a and b denote significant differences (*P*<0.05) compared with the untreated control and CDDP-treated groups, respectively.

As represented in Figure 2, the i.p. injection of CDDP evoked noticeable rises (*P*<0.05) in the renal levels of MDA and NO together with decline (*P*<0.05) in GSH levels in the renal tissue in respect to the controls. On the other hand, Tur, Tur-SeNPs, or NAC treatment reduced (*P*<0.05) markedly MDA and NO levels and increased (*P*<0.05) the renal GSH contents in CDDP-treated group. It is noteworthy that the values of the abovementioned parameters in Tur-SeNPs-administered group were close to those of the control group.

**Figure 3:**
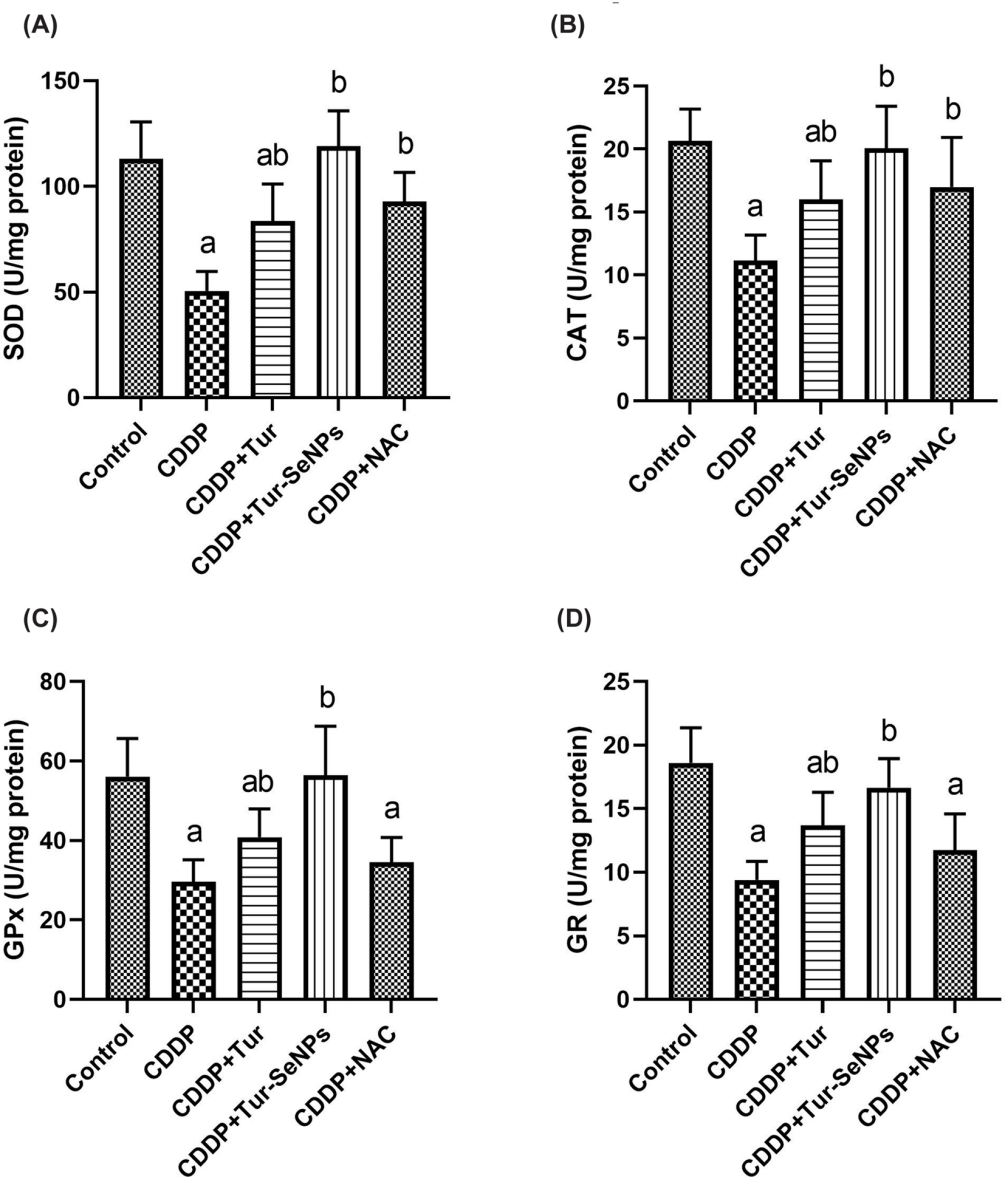
Effects of orally administered turmeric (Tur), turmeric-loaded selenium nanoparticles (Tur-SeNPs), or N-acetyl cysteine (NAC) on the antioxidant enzymatic activities of (A) SOD, (B) CAT, (C) GPx, and (D) GR in the kidney of cisplatin (CDDP)-injected mice Data are expressed as mean ± SD (*n*=7). a and b denote significant differences (*P*<0.05) compared with the untreated control and CDDP-treated groups, respectively.

Concerning the antioxidant enzymatic activities, a single dose of injected CDDP induced marked suppression (*P*<0.05) in renal SOD, GR, GPx, and CAT when compared with the control group. The therapeutic administration of Tur, Tur-SeNPs or NAC enhanced the antioxidant response in mouse kidneys of CDDP group as indicated by remarkable rise (*P*<0.05) in the antioxidant enzymatic activities. Similar antioxidant potential was also observed by NAC in CDDP-injured kidneys. Notably, Tur loaded SeNPs was successful to restore these antioxidant biomarkers when compared to the sole administration of Tur, indicating its stronger antioxidant capacity (Figure 3).

**Figure 4:**
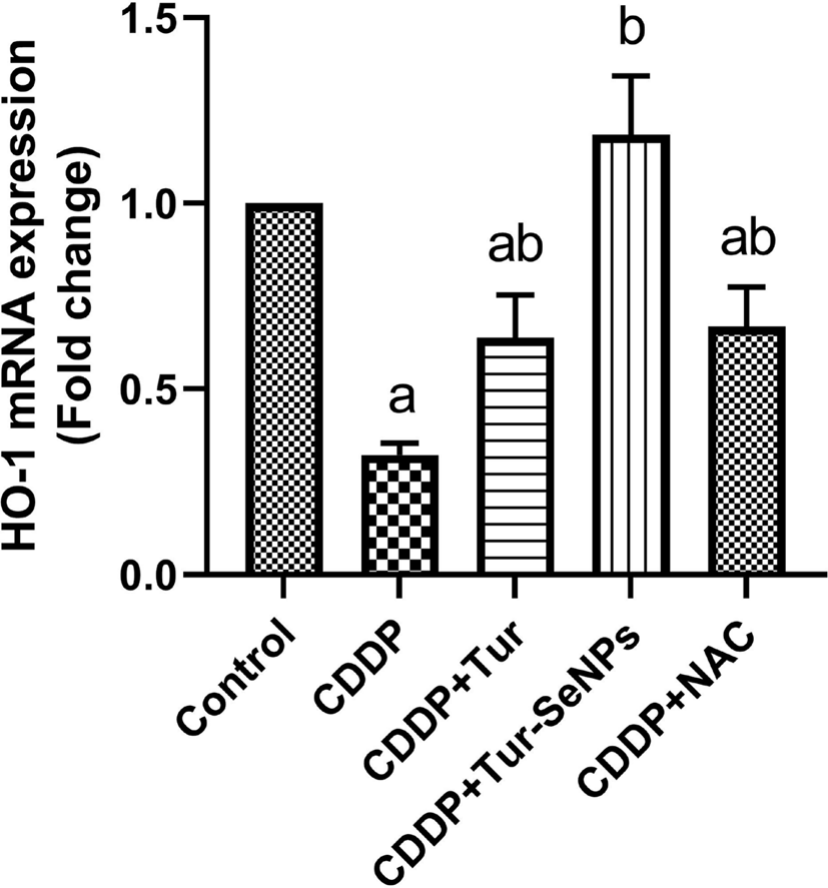
Effects of orally administered turmeric (Tur), turmeric-loaded selenium nanoparticles (Tur-SeNPs), or N-acetyl cysteine (NAC) on the mRNA gene expression of hemeoxygenase (HO-1) in the kidney of cisplatin (CDDP)-injected mice Data are expressed as mean ± SD (*n*=3 in duplicate). The mRNA levels were quantified with GAPDH as an internal control. a and b denote significant differences (*P*<0.05) compared with the untreated control and CDDP-treated groups, respectively.

Using qRT-PCR technique, we investigated the effect of Tur alone or conjugated with SeNPs on the transcriptional level of HO-1 in the renal tissue of treated mice (Figure 4). CDDP treatment caused significant down-regulation (*P*<0.05) in the relative mRNA levels of HO-1 compared with the control values. In contrast, administration of Tur, Tur-SeNPs, or NAC to mice exposed to CDDP meaningfully up-regulated (*P*<0.05) HO-1 gene expression when compared with the group with CDDP challenge.

### Effects of Tur and Tur-SeNPs on renal inflammation of CDDP-injected mice

**Figure 5:**
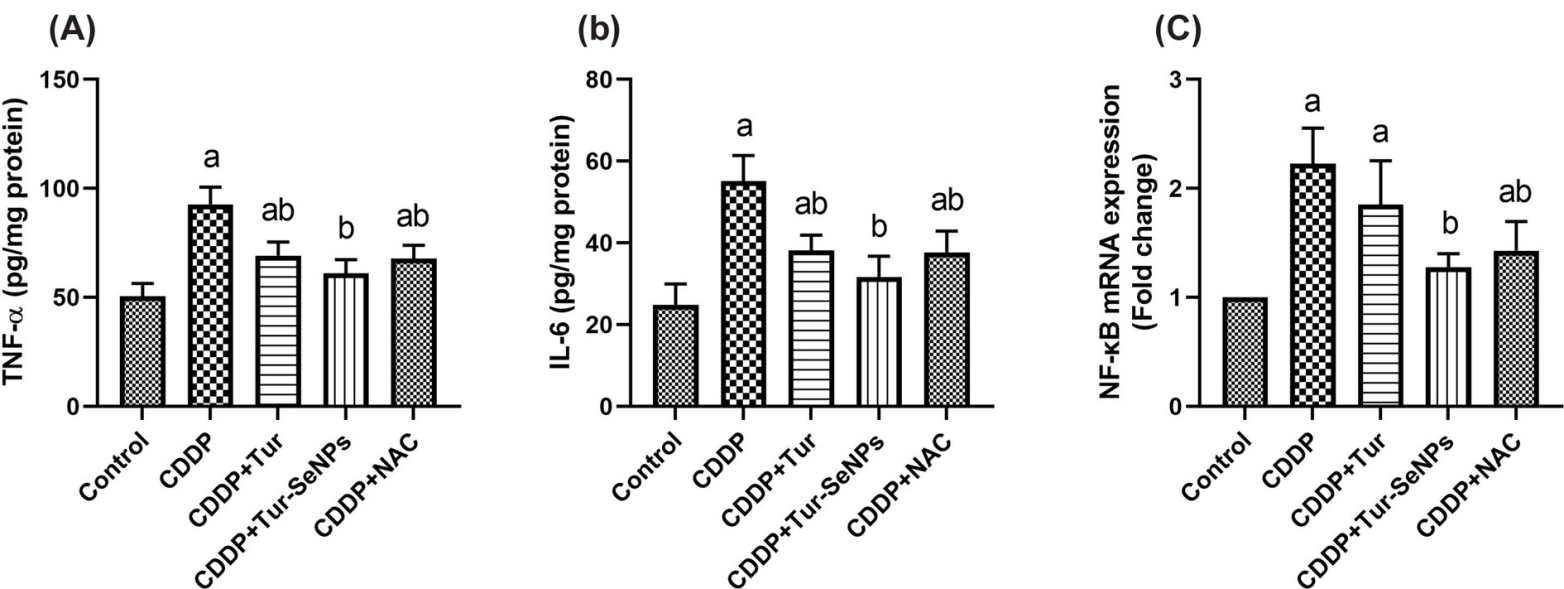
Effects of orally administered turmeric (Tur), turmeric-loaded selenium nanoparticles (Tur-SeNPs), or N-acetyl cysteine (NAC) on the renal levels of (A) IL-6 and (B) TNF-α as well as (C) the mRNA gene expression of nuclear factor kappa-B (NF-κB) in the kidney of cisplatin (CDDP)-injected mice Data are expressed as mean ± SD (*n*=7). The mRNA levels for NF-κB were quantified with GAPDH as an internal control. a and b denote significant differences (*P*<0.05) compared with the untreated control and CDDP-treated groups, respectively.

In order to assess the renal inflammatory reaction in response to different treatments, pro-inflammatory cytokine levels and NF-κB mRNA expression were measured (Figure 5). CDDP injection induced an obvious upsurge (*P*<0.05) in the renal pro-inflammatory indices (TNF-α and IL-6) compared with the controls. However, treatment with Tur, Tur-SeNPs, or NAC significantly mitigated (*P*<0.05) such increases in the tested kidney cytokines mediated by CDDP challenge.

In addition, the mice exposed to CDDP had a noticeable up-regulated (*P*<0.05) gene expression level of NF-κB compared with the untreated group. On the other side, CDDP-injected mice and treated with Tur-SeNPs or NAC showed a remarkable lower gene transcription levels (*P*<0.05) of NF-κB than those in the CDDP group. This reduction was near to the control value in Tur-SeNPs-treated group. No significant changes were noticed in expression of this transcriptional factor upon the comparison between CDDP and CDDP+Tur-treated groups.

### Effects of Tur and Tur-SeNPs on renal apoptotic markers of CDDP-injected mice

**Figure 6:**
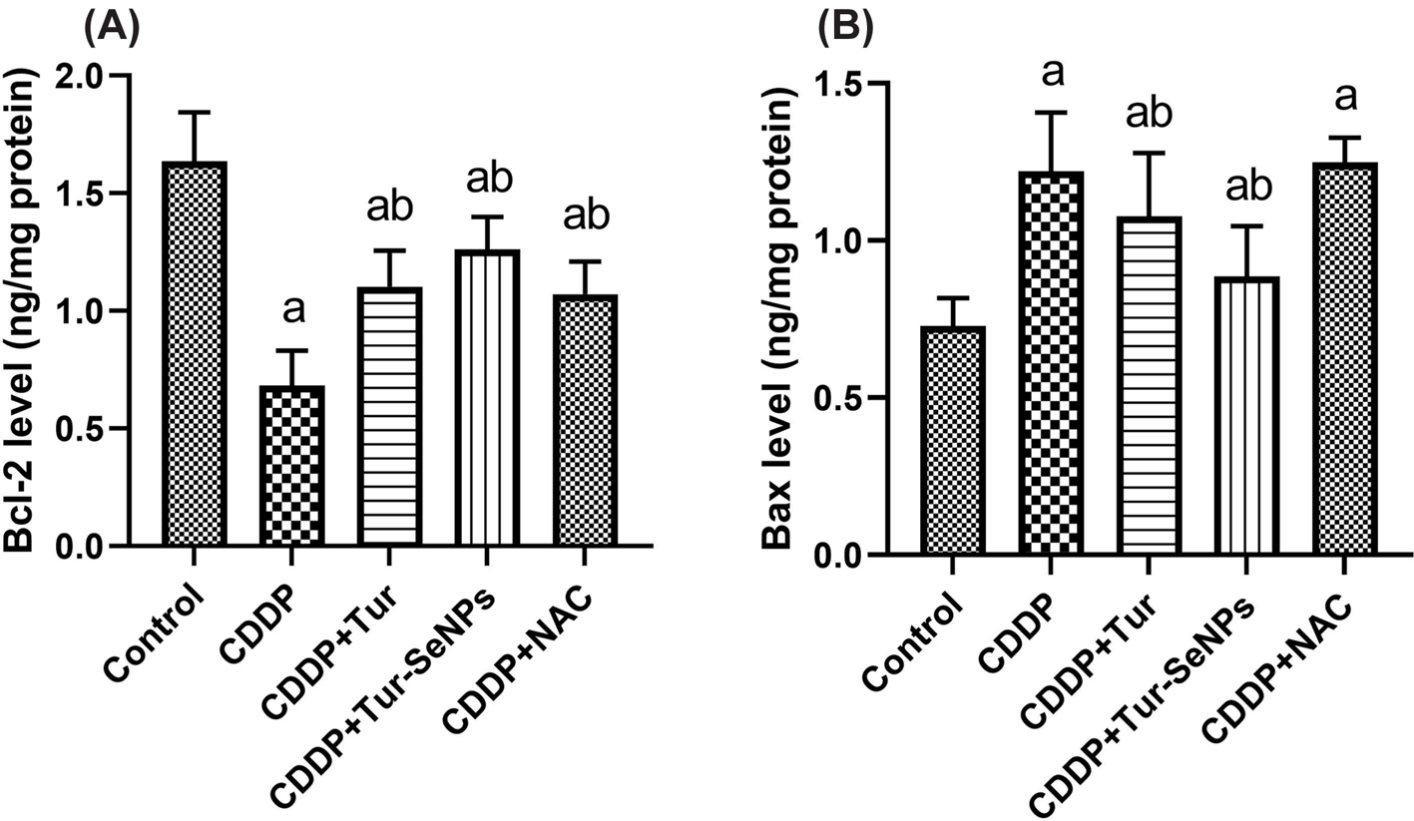
Effects of orally administered turmeric (Tur), turmeric-loaded selenium nanoparticles (Tur-SeNPs), or N-acetyl cysteine (NAC) on the levels of renal apoptotic markers [(A) Bcl-2 and (B) Bax] in cisplatin (CDDP)-injected mice Data are expressed as mean ± SD (*n*=7). a and b denote significant differences (*P*<0.05) compared with the untreated control and CDDP-treated groups, respectively.

Apoptotic cell death plays an important role in CDDP-induced nephrotoxicity and mediated by the interplay between pro-apoptotic protein (Bax) and anti-apoptotic proteins (Bcl-2). We observed in our study outstanding decreases (*P*<0.05) in renal Bcl-2 levels, together with considerable increase (*P*<0.05) in renal Bax levels in the CDDP group as compared with the untreated group. Nevertheless, Tur or Tur-SeNPs-treated groups showed notable upsurges (*P*<0.05) in Bcl-2 levels and substantial decline (*P*<0.05) in Bax levels in comparison with CDDP group. Furthermore, the treatment of CDDP toxicity with NAC evoked marked increases (*P*<0.05) in Bcl-2 levels without any significant changes in Bax levels (Figure 6).

### Effect of Tur or Tur-SeNPs on renal histological features of CDDP-injected mice

**Figure 7:**
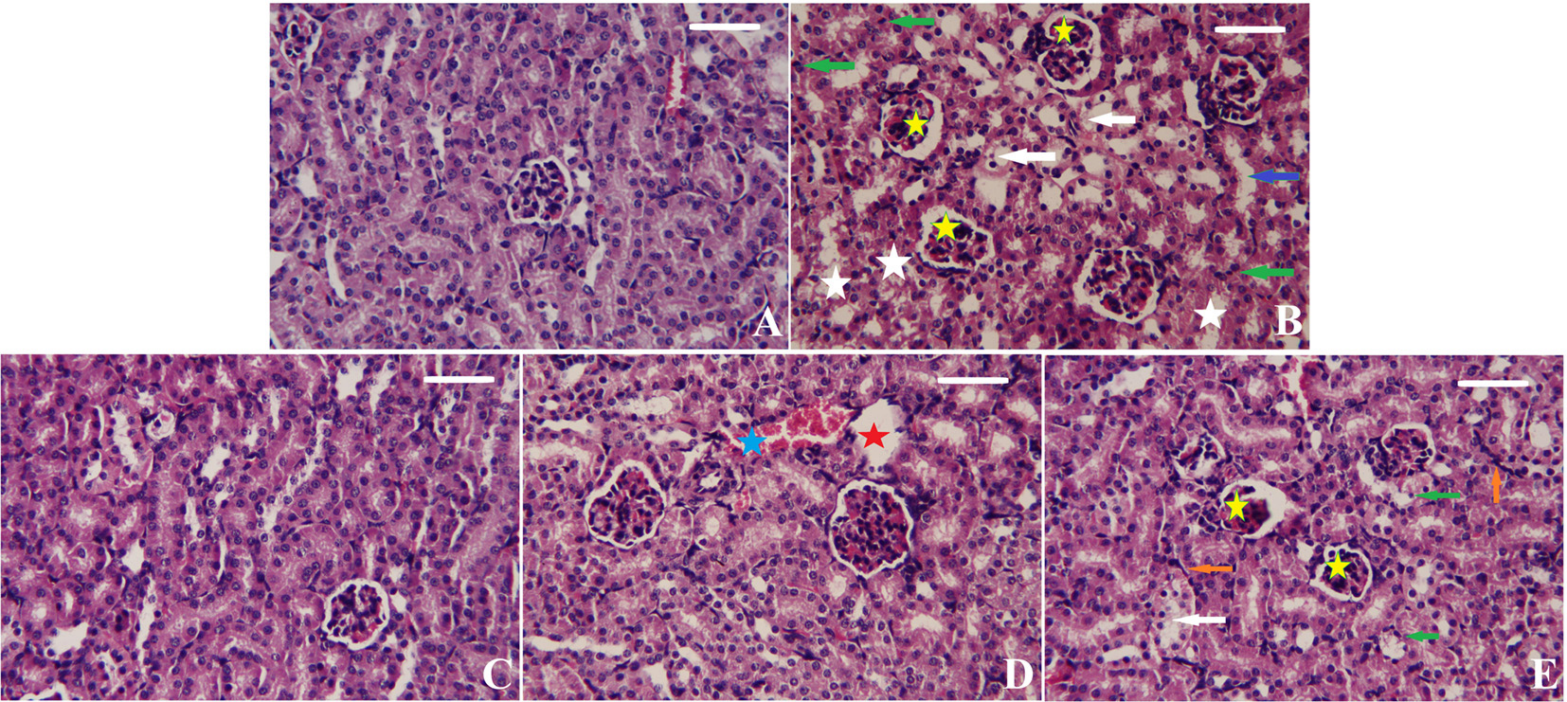
Effects of orally administered turmeric (Tur), turmeric-loaded selenium nanoparticles (Tur-SeNPs), or N-acetyl cysteine (NAC) on the renal histomorphological features of cisplatin (CDDP)-injected mice (**A**) Photomicrograph of the renal tissue of the control group showing healthy kidney structure. (**B**) Photomicrograph of the renal tissue of mice treated with cisplatin (CDDP) showing shrunken glomeruli (yellow stars), cellular degeneration (white arrows), necrotic tubules (white stars), apoptosis (green arrows), and dilated tubules (blue arrows). (**C**) Photomicrograph of the renal tissue of CDDP-injected mice treated with turmeric (Tur) showing normal kidney structure. (**D**) Photomicrograph of the renal tissue of CDDP-injected mice treated with Tur-SeNPs showing damaged glomeruli (red stars) and hemorrhage (blue stars). (**E**) Photomicrograph of the renal tissue of CDDP-injected mice treated with NAC showing shrunken glomeruli (yellow stars), cellular degeneration (white arrows), apoptosis (green arrows), and inflammatory cell infiltration (orange arrows). Sections were stained with hematoxylin and eosin (× 400; scale bar = 100 µm).

The kidney sections from different groups are shown in Figure 7. The control group showed normal kidney morphology of renal glomeruli and tubules without any observed pathological lesions (Figure 7A). On the contrary, CDDP injection resulted in remarkable shrunken glomeruli, cellular degeneration, necrotic tubules, apoptosis, and dilated tubules (Figure 7B). Pre-treatment with Tur or Tur-SeNPs significantly ameliorated these pathological changes in mice kidneys challenged by CDDP (Figures 7C and 4D). In addition, a significant improvement in the kidney histoarchitecture was noticed in CDDP+NAC group (Figure 7E); however, damaged glomeruli, apoptotic cells and inflammatory cell infiltration were seen.

## Discussion

It has been reported that the discerning accumulation of CDDP in the renal cortex is accompanied by severe nephrotoxicity that is accredited to the functional properties of the kidney to eliminate anti-tumor drugs and metabolites effectively [5,7]. Following a single administration of CDDP, serum urea and creatinine as well as the kidney index increased, as compared with the normal group. As being the waste end products of protein metabolism that are excreted by the kidneys, increases in these metabolites advocate renal failure due to damage of renal glomeruli and tubules [26,46–48]. Noteworthy, renal histopathological screening showed focal areas of necrosis, and tubular epithelium as well as damaged glomeruli. Another evidence to consider is the marked upsurge in serum NGAL and KIM-1 levels after CDDP injection that confirmed severe renal injury because they are sensitive biomarkers in the initiation of kidney injury [49,50]. These findings are in agreement with former reports [4,7,50]. In a clinical trial, urinary Kim-1, Cystatin C, and NGAL displayed substantial increases earlier than creatinine and urea in patients receiving platinum-based therapy [51]. An *in vitro* study performed by Oh and colleagues also revealed that treatment with CDDP increased the levels of NGAL and Kim-1 in treated kidney proximal tubular cells [49].

On the other hand, this impairment in kidney function and the renal histological alterations evoked by CDDP was markedly restored in the groups that received Tur or Tur-SeNPs, which revealed their nephroprotective potential. It was formerly observed that turmeric-extracted curcumin, in a dose-dependent manner, decreased serum urea and creatinine levels in a rat model of arsenic-induced nephrotoxicity [18]. Furthermore, El-Gizawy et al. [19] reported that curcumin nanoparticles counteracted the increases in serum levels of urea, uric acid, and creatinine mediated by CDDP injection in intoxicated rats. Co-treatment with curcumin nanoparticles offered marked decreases in renal function markers as well as Kim-1 and lipocalin-2 in hydroxyapatite nanoparticle-exposed rats [52]. Similar findings were also noticed by Anwar and colleagues in rabbits receiving curcumin loaded chitosan nanoparticles to protect against cypermethrin induced nephrotoxicity [53]. Furthermore, the administration of SeNPs was formerly reported to lessen the increased renal function biomarkers in acute kidney injury [26], diabetes [29], and cadmium toxicity [54] in rat models. In addition, previous investigations confirmed the nephroprotective effect of green synthetized SeNPs. Zahran et al. [55] found significant improvements in renal function in nicotine-exposed rats and concomitantly administered with *ginger Zingiber officinale*. Likewise, pre-treatment with SeNPs synthesized using *Spermacoce hispida* attenuated the acetaminophen-induced elevated renal injury markers. Overall, these results showed that Tur-SeNPs exerted a noteworthy protective effect against CDDP-induced nephrotoxicity and this effect was probably associated with their antioxidant properties.

It is an undeniable fact that overgeneration of ROS has a vital role in CDDP-mediated renal damage. In harmony with previous reports [4,5,7], our study confirmed a pronounced oxidative stress in renal tissue after CDDP injection in male mice. CDDP was reported to react with cellular proteins and thiol containing molecules that result in a decrease in the antioxidant defense [1]. Our study also revealed a marked down-regulation of HO-1 gene expression in CDDP-injected group. Under an undesirable condition of oxidative stress, Keap1 triggers the activation of Nrf2 and its downstream-regulated genes [56]. Nrf2 regulates the induction of detoxification enzymes and antioxidants such as HO-1 for scavenging of deleterious radicals and maintenance of cellular homeostasis [57,58]. Recent articles, in accordance with our study, have reported that CDDP results in down-regulation of Nrf2 and its related antioxidant enzymes [7,50].

Interestingly, Tur or Tur-SeNPs treatment can effectively inhibit CDDP-induced renal oxidative stress suggesting their protective effect that is associated with their antioxidant properties. In the same regard, Pakfetrat et al. [59] found notable increases in the activities of GPX, GR, and CAT with decreases in MDA levels in the plasma of hemodialysis patients receiving Tur regularly for eight weeks. As well, concomitant administration of curcumin with colistin displayed remarkable renoprotective action by augmenting the antioxidant biomarkers (CAT and GSH) and reducing the MDA levels [60]. These outcomes are in accordance with previous findings where Tur or curcumin reduced ROS generation and enhanced antioxidant mediators [16,61,62]. This effect of Tur might be attributed to the abundance of phenolic and flavonoid compounds, which could successfully inhibit the oxidative stress associated with CDDP injection [61]. Moreover, curcumin in nanoforms were able to alleviate the renal oxidative injury induced by hydroxyapatite nanoparticles in treated male rats [63]. The authors endorsed the ROS scavenging effect of turmeric-extracted curcumin by the electron transfer and donation of hydrogen atoms. Moreover, oral SeNPs administration restored the antioxidant balance in the renal tissue in a rat model of acute kidney injury [64]. Supplementation with SeNPs elicited a remarkable antioxidant efficacy against diabetic nephropathy in pregnant female rats [29]. Similar outcomes were also reported by Sadek and colleagues in cadmium chloride [54] and vancomycin [30]-mediated renal stresses. The ability of selenium to quench hydrogen peroxide radicals refers to its position as a selenocysteine residue in the active site of selenoproteins, including GPx [65,66]. Previous studies had reported that green synthetized SeNPs with phytochemicals offered a distinguished antioxidant power than that of SeNPs alone [26,35]. Likewise, biosynthesized SeNPs with the endophytic fungus *Fusarium oxysporum* evoked marked reductions in the generation of free radicals and MDA in the hepatic, renal and cardiac tissues of doxorubicin-injected mice [33]. Synthetized SeNPs using *Spermacoce hispida* were reported to accomplish significant antioxidant effects against hepatorenal toxicity accompanied by acetaminophen exposure [35]. This is due to the synergistic effect of Tur and SeNPs to guard the rat kidney against CDDP toxicity through increasing endogenous antioxidants and protecting mitochondrial functions. Besides, recent investigations have demonstrated the contribution of Nrf2/HO-1 pathway in the palliative effect of Tur against cellular oxidative stress [62,67]. Lui et al. [68] found that curcumin intervention evoked noteworthy rises in the levels of gene and protein expression of Nrf2, NQO1, HO-1, and Keap1 in renal injury induced by aristolochic acid in rats. In addition, SeNPs were found to protect against epilepsy-mediated oxidative challenge via the up-regulation of Nrf2 and HO-1 [25]. The activation of Nrf2 and its downstream genes was also observed after the administration of biogenic SeNPs [26,69]. Our results coherently revealed indicated that Tur-SeNPs supplementation might enhance the nephritic Nrf2 and its downstream gene HO-1 that counteracted CDDP-induced renal impairment in mice.

Oxidative stress is well known to boost the production of inflammatory mediators that strongly involved in the pathophysiology of CDDP-induced renal damage [4,5]. Among these mediators, TNF-α is recognized as a key mediator in the expression of inflammatory cytokines and recruitment of immune cells in the renal tissue after CDDP challenge [70]. In this study, CDDP injection significantly increased the levels of TNF-α and IL-6 as well as the mRNA expression of NF-κB in the mouse kidneys. NF-κB is the hub of many signaling pathways and its activation enhances the transcription of various pro-inflammatory cytokines [57,71]. It was documented that the activation of NF-κB is the main dedicator of renal inflammation under CDDP challenge [5,7]. Furthermore, the activation of NF-κB stimulates the transcription of other inflammatory-mediated genes such as COX-2 and iNOS [3].

On the other side, a remarkable anti-inflammatory effect was observed in CDDP-injected group and treated with Tur or Tur-SeNPs that may endorse for the ability to suppress the pro-inflammatory cytokines in renal tissue. Our findings are in agreement with former reports that validated the anti-inflammatory potential of Tur [19,52,60,67]. In fact, a clinical study reported that curcumin supplementation for 12 weeks markedly reduced plasma TNF-α levels in patients with chronic kidney disease undergoing hemodialysis [20]. Furthermore, urinary concentrations of MCP-1 and TGF-β were significantly reduced upon treatment with Tur extract, suggesting its antagonistic action against doxorubicin-induced kidney tissue inflammation [15]. Moreover, the anti-inflammatory potential of SeNPs has been previously recorded [52,55,64]. The treatment with SeNPs coated with lycopene markedly halted the rise in the levels of TNF-α, IL-1β, IL-6, and gene expression of nitric oxide synthase in acute kidney injury-mediated renal inflammation [26]. Our study suggests that this mitigating effect of Tur-SeNPs on renal inflammation has been accredited to the involvement of NF-κB pathway. Xu et al. [67] have demonstrated that curcumin noticeably reversed the phosphorylation of NF-κB in kidney tissue of arsenic-exposed mice that subsequently inhibited the excess secretion of inflammatory cytokines. Likewise, curcumin NPs down-regulated the protein expression levels of NF-κB-p65 and inflammatory cytokines in a rat model of cerebral ischemia [72]. Zaghloul et al. [73] found that SeNPs decreased the diabetic nephropathy-associated rise in IL-6, TNF-α, and NF-κB levels in rat kidney.

Furthermore, both inflammatory and oxidative pathways are responsible for the mitochondrial damage, which triggers the apoptotic pathway in CDDP-evoked renal injury [8]. This study, in consistence with previous studies [3,6,8], showed marked increased renal Bax levels while diminished Bcl-2 levels in CDDP-injected group. Through the induction of excess ROS, CDDP can activate proapoptotic proteins, which change the permeability of the mitochondrial membrane and stimulate the release of cytochrome *c*. Next, the cytoplasmic translocation of cytochrome *c* results in activation of caspases and cellular apoptosis [74]. On the other hand, this apoptotic damage was notably reversed by Tur-SeNPs administration. This is in harmony with earlier studies that declared the anti-apoptotic potential of Tur and SeNPs [26,30,60]. Curcumin treatment effectively reduced the apoptotic index in the proximal renal tubules in zinc oxide nanoparticles-exposed rats [16]. In addition, biogenic SeNPs synthesized using *Moringa oleifera* leaf extract antagonized renal apoptosis in melamine-exposed rats via down-regulation of the mRNA expressions of Bax, caspase-3, Bcl2, Fas, and FasL [34]. Similar anti-apoptotic effect was reported by another study using starch-stabilized Se-NPs [75]. This activity may endorse for scavenging of free radicals efficiently and impeding the apoptotic mitochondrial pathways.

## Conclusions

In brief, our study sheds light on the reno-protective impact of Tur extract alone or conjugated with SeNPs against CDDP-induced nephrotoxicity in a mouse model. Tur-SeNPs effectively restored CDDP-mediated increases in serum levels of kidney function indices (urea, creatinine, KIM-1, and NGAL). Moreover, Tur-SeNPs exerted its renal protection via the interplay of oxidative damage, inflammatory reaction, and apoptotic cell death. The nanoformulated particles enhanced the renal antioxidant defense as demonstrated by decreases in MDA and NO besides increases in GSH levels and antioxidant enzymatic activity. The molecular results revealed that Tur-SeNPs down-regulated the expression of NF-κB and up-regulated the expression of HO-1that supported their antioxidative and anti-inflammatory capacities. Tur-SeNPs also decreased noticeably the renal inflammatory cytokines and apoptotic markers together with restoring of the healthy renal histoarchitecture. The existing findings developed SeNPs synthetized with Tur extract as a new therapeutic possibility to guard against CDDP-induced devastating kidney damage.

## Institutional Review Board Statement

The experiment as well as the procedures and methodologies, were designed in compliance with the standards of Helwan University's Institutional Animal Care and Use Committee (IACUC) (approval no HU2021/Z/AES0921-02).

## Data Availability

We confirm that all original raw data is available at the time of submission. As per the Data Policy, these data will be stored for a minimum of 10 years and will be made available to the Editorial Office, Editors and readers upon request.

## Abbreviations

CAT: catalase
CDDP: cisplatin
GPx: glutathione peroxidase
GR: glutathione reductase
NAC: N-acetyl cysteine
ROS: reactive oxygen species
SOD: superoxide dismutase
Tur: turmeric

## References

[1] Mahran Y.F. (2020) New insights into the protection of growth hormone in cisplatin-induced nephrotoxicity: the impact of IGF-1 on the Keap1-Nrf2/HO-1 signaling. Life Sci. 253, 117581 10.1016/j.lfs.2020.117581 32209424

[2] Lin W.-H., Jiang W.-P., Chen C.-C., Lee L.-Y., Tsai Y.-S., Chien L.-H. et al. (2022) Renoprotective Effect of Pediococcus acidilactici GKA4 on cisplatin-induced acute kidney injury by mitigating inflammation and oxidative stress and regulating the MAPK, AMPK/SIRT1/NF-κB, and PI3K/AKT pathways. Nutrients 14, 2877 10.3390/nu14142877 35889833 PMC9323173

[3] Michel H.E. and Menze E.T. (2019) Tetramethylpyrazine guards against cisplatin-induced nephrotoxicity in rats through inhibiting HMGB1/TLR4/NF-κB and activating Nrf2 and PPAR-γ signaling pathways. Eur. J. Pharmacol. 857, 172422 10.1016/j.ejphar.2019.172422 31152701

[4] Jing T., Liao J., Shen K., Chen X., Xu Z., Tian W. et al. (2019) Protective effect of urolithin a on cisplatin-induced nephrotoxicity in mice via modulation of inflammation and oxidative stress. Food Chem. Toxicol. 129, 108–114 10.1016/j.fct.2019.04.031 31014901

[5] Liu S., Zhang X. and Wang J. (2020) Isovitexin protects against cisplatin-induced kidney injury in mice through inhibiting inflammatory and oxidative responses. Int. Immunopharmacol. 83, 106437 10.1016/j.intimp.2020.106437 32222637

[6] Domitrović R., Cvijanović O., Pernjak-Pugel E., Škoda M., Mikelić L. and Crnčević-Orlić Ž. (2013) Berberine exerts nephroprotective effect against cisplatin-induced kidney damage through inhibition of oxidative/nitrosative stress, inflammation, autophagy and apoptosis. Food Chem. Toxicol. 62, 397–406 10.1016/j.fct.2013.09.003 24025684

[7] Wahdan S.A., Azab S.S., Elsherbiny D.A. and El-Demerdash E. (2019) Piceatannol protects against cisplatin nephrotoxicity via activation of Nrf2/HO-1 pathway and hindering NF-κB inflammatory cascade. Naunyn Schmiedeberg’s Arch. Pharmacol. 392, 1331–1345 10.1007/s00210-019-01673-8 31197431

[8] Zhang Y., Chen Y., Li B., Ding P., Jin D., Hou S. et al. (2020) The effect of monotropein on alleviating cisplatin-induced acute kidney injury by inhibiting oxidative damage, inflammation and apoptosis. Biomed. Pharmacother. 129, 110408 10.1016/j.biopha.2020.110408 32574971

[9] Streyczek J., Apweiler M., Sun L. and Fiebich B.L. (2022) Turmeric Extract (Curcuma longa) mediates anti-oxidative effects by reduction of nitric oxide, iNOS Protein-, and mRNA-Synthesis in BV2 microglial cells. Molecules 27, 10.3390/molecules27030784 35164047 PMC8840760

[10] Uchio R., Kawasaki K., Okuda-Hanafusa C., Saji R., Muroyama K., Murosaki S. et al. (2021) Curcuma longa extract improves serum inflammatory markers and mental health in healthy participants who are overweight: a randomized, double-blind, placebo-controlled trial. Nutr. J. 20, 91 10.1186/s12937-021-00748-8 34774052 PMC8590273

[11] Yuliani S., Mustofa and Partadiredja G. (2019) The neuroprotective effects of an ethanolic turmeric (Curcuma longa L.) extract against trimethyltin-induced oxidative stress in rats. Nutr. Neurosci. 22, 797–804 10.1080/1028415X.2018.1447267 29513140

[12] Lee H.Y., Kim S.W., Lee G.H., Choi M.K., Jung H.W., Kim Y.J. et al. (2016) Turmeric extract and its active compound, curcumin, protect against chronic CCl4-induced liver damage by enhancing antioxidation. BMC Complement. Altern. Med. 16, 316 10.1186/s12906-016-1307-6 27561811 PMC5000414

[13] Uchio R., Higashi Y., Kohama Y., Kawasaki K., Hirao T., Muroyama K. et al. (2017) A hot water extract of turmeric (Curcuma longa) suppresses acute ethanol-induced liver injury in mice by inhibiting hepatic oxidative stress and inflammatory cytokine production. J. Nutritional Sci. 6, e3 10.1017/jns.2016.43 PMC5465857 28620478

[14] Moghadam A.R., Tutunchi S., Namvaran-Abbas-Abad A., Yazdi M., Bonyadi F., Mohajeri D. et al. (2015) Pre-administration of turmeric prevents methotrexate-induced liver toxicity and oxidative stress. BMC Complement. Altern. Med. 15, 246 10.1186/s12906-015-0773-6 26199067 PMC4511036

[15] Russo E.R., Facincani I., Nakazato K.C., Coimbra T.M., Crevelin E.J., Pereira A.M.S. et al. (2018) Oral administration of powdered dried rhizomes of Curcuma longa L. (turmeric, Zingiberaceae) is effective in the treatment of doxorubicin-induced kidney injury in rats. Phytotherapy Res.: PTR 32, 2408–2416 10.1002/ptr.6176 30109739

[16] Heidai-Moghadam A., Khorsandi L. and Jozi Z. (2019) Curcumin attenuates nephrotoxicity induced by zinc oxide nanoparticles in rats. Environmental Sci. Pollution Res. 26, 179–187 10.1007/s11356-018-3514-9 30387060

[17] Kim K.S., Lim H.-J., Lim J.S., Son J.Y., Lee J., Lee B.M. et al. (2018) Curcumin ameliorates cadmium-induced nephrotoxicity in Sprague-Dawley rats. Food Chem. Toxicol. 114, 34–40 10.1016/j.fct.2018.02.007 29421648

[18] Ishaq A., Gulzar H., Hassan A., Kamran M., Riaz M., Parveen A. et al. (2021) Ameliorative mechanisms of turmeric-extracted curcumin on arsenic (As)-induced biochemical alterations, oxidative damage, and impaired organ functions in rats. Environment. Sci. Pollution Res. 28, 66313–66326 10.1007/s11356-021-15695-4 34331650

[19] El-Gizawy M.M., Hosny E.N., Mourad H.H., Razik A.-E. and Amira N. (2020) Curcumin nanoparticles ameliorate hepatotoxicity and nephrotoxicity induced by cisplatin in rats. Naunyn Schmiedeberg’s Arch. Pharmacol. 393, 1941–1953 10.1007/s00210-020-01888-0 32447466

[20] Alvarenga L., Cardozo L., Da Cruz B.O., Paiva B.R., Fouque D. and Mafra D. (2022) Curcumin supplementation improves oxidative stress and inflammation biomarkers in patients undergoing hemodialysis: a secondary analysis of a randomized controlled trial. Int. Urol. Nephrol. 54, 2645–2652 10.1007/s11255-022-03182-9 35347555

[21] Zou P., Zhang J., Xia Y., Kanchana K., Guo G., Chen W. et al. (2015) ROS generation mediates the anti-cancer effects of WZ35 via activating JNK and ER stress apoptotic pathways in gastric cancer. Oncotarget 6, 5860–5876 10.18632/oncotarget.3333 25714022 PMC4467407

[22] Venkatas J., Daniels A. and Singh M. (2022) The potential of curcumin-capped nanoparticle synthesis in cancer therapy: a green synthesis approach. Nanomaterials (Basel) 12, 10.3390/nano12183201 36144994 PMC9502936

[23] Aggarwal M.L., Chacko K.M. and Kuruvilla B.T. (2016) Systematic and comprehensive investigation of the toxicity of curcuminoid-essential oil complex: a bioavailable turmeric formulation. Mol. Med. Rep. 13, 592–604 10.3892/mmr.2015.4579 26648561 PMC4686098

[24] Jantawong C., Priprem A., Intuyod K., Pairojkul C., Pinlaor P., Waraasawapati S. et al. (2021) Curcumin-loaded nanocomplexes: acute and chronic toxicity studies in mice and hamsters. Toxicol. Rep. 8, 1346–1357 10.1016/j.toxrep.2021.06.021 34277359 PMC8267493

[25] Yuan X., Fu Z., Ji P., Guo L., Al-Ghamdy A.O., Alkandiri A. et al. (2020) Selenium nanoparticles pre-treatment reverse behavioral, oxidative damage, neuronal loss and neurochemical alterations in pentylenetetrazole-induced epileptic seizures in mice. Int. J. Nanomed. 15, 6339 10.2147/IJN.S259134 PMC7455605 32922005

[26] Al-Brakati A., Alsharif K.F., Alzahrani K.J., Kabrah S., Al-Amer O., Oyouni A.A. et al. (2021) Using green biosynthesized lycopene-coated selenium nanoparticles to rescue renal damage in glycerol-induced acute kidney injury in rats. Int. J. Nanomed. 16, 4335–4349 10.2147/IJN.S306186 PMC8254550 34234429

[27] Khubulava S., Chichiveishvili N., Shavshishvili N., Mulkijanyan K., Khodeli N., Jangavadze M. et al. (2019) Effect of high dose of selenium nanoparticles on alimentary tract in rodents. J. Nanomed. Nanotechnol. 10, 531 10.35248/2157-7439.19.10.531

[28] Lesnichaya M., Shendrik R., Titov E. and Sukhov B. (2020) Synthesis and comparative assessment of antiradical activity, toxicity, and biodistribution of kappa-carrageenan-capped selenium nanoparticles of different size: in vivo and in vitro study. IET Nanobiotechnol. 14, 519–526 10.1049/iet-nbt.2020.0023 32755962 PMC8676537

[29] Alhazza I.M., Ebaid H., Omar M.S., Hassan I., Habila M.A., Al-Tamimi J. et al. (2022) Supplementation with selenium nanoparticles alleviates diabetic nephropathy during pregnancy in the diabetic female rats. Environment. Sci. Pollution Res. 29, 5517–5525 10.1007/s11356-021-15905-z 34420167

[30] Mehanna E.T., Khalaf S.S., Mesbah N.M., Abo-Elmatty D.M. and Hafez M.M. (2022) Anti-oxidant, anti-apoptotic, and mitochondrial regulatory effects of selenium nanoparticles against vancomycin induced nephrotoxicity in experimental rats. Life Sci. 288, 120098 10.1016/j.lfs.2021.120098 34715137

[31] Al-Brakati A., Alsharif K.F., Alzahrani K.J., Kabrah S., Al-Amer O., Oyouni A.A. et al. (2021) Using green biosynthesized lycopene-coated selenium nanoparticles to rescue renal damage in glycerol-induced acute kidney injury in rats. Int. J. Nanomed. 16, 4335 10.2147/IJN.S306186 PMC8254550 34234429

[32] Mohamed K.M., Abdelfattah M.S., El-khadragy M., Al-Megrin W.A., Fehaid A., Kassab R.B. et al. (2023) Rutin-loaded selenium nanoparticles modulated the redox status, inflammatory, and apoptotic pathways associated with pentylenetetrazole-induced epilepsy in mice. Green Process Synth. 12, 20230010 10.1515/gps-2023-0010

[33] Khan M.A., Singh D., Arif A., Sodhi K.K., Singh D.K., Islam S.N. et al. (2022) Protective effect of green synthesized selenium nanoparticles against doxorubicin induced multiple adverse effects in Swiss albino mice. Life Sci. 305, 120792 10.1016/j.lfs.2022.120792 35817167

[34] Abu-Zeid E.H., Abdel Fattah D.M., Arisha A.H., Ismail T.A., Alsadek D.M., Metwally M.M.M. et al. (2021) Protective prospects of eco-friendly synthesized selenium nanoparticles using Moringa oleifera or Moringa oleifera leaf extract against melamine induced nephrotoxicity in male rats. Ecotoxicol. Environ. Saf. 221, 112424 10.1016/j.ecoenv.2021.112424 34174736

[35] Krishnan V., Loganathan C. and Thayumanavan P. (2019) Green synthesized selenium nanoparticles using Spermacoce hispida as carrier of s-allyl glutathione: to accomplish hepatoprotective and nephroprotective activity against acetaminophen toxicity. Artificial Cells, Nanomed. Biotechnol. 47, 56–63 10.1080/21691401.2018.1543192 30669860

[36] Serairi Beji R., Ben Mansour R., Bettaieb Rebey I., Aidi Wannes W., Jameleddine S., Hammami M. et al. (2019) Does Curcuma longa root powder have an effect against CCl(4)-induced hepatotoxicity in rats: a protective and curative approach. Food Sci. Biotechnol. 28, 181–189 10.1007/s10068-018-0449-3 30815309 PMC6365324

[37] Abdel Moneim A.E., Othman M.S. and Aref A.M. (2014) Azadirachta indica attenuates cisplatin-induced nephrotoxicity and oxidative stress. BioMed. Res. Int. 2014, 10.1155/2014/647131 25162019 PMC4137610

[38] Al-Quraishy S., Dkhil M.A., Abdel-Gaber R., Zrieq R., Hafez T.A., Mubaraki M.A. et al. (2020) Myristica fragrans seed extract reverses scopolamine-induced cortical injury via stimulation of HO-1 expression in male rats. Environ. Sci. Pollut. Res. Int. 27, 12395–12404 10.1007/s11356-020-07686-8 31993909

[39] Yagi K. (1998) Simple assay for the level of total lipid peroxides in serum or plasma. Free radical and antioxidant protocols, pp. 101–106, Springer 10.1385/0-89603-472-0:101 9921519

[40] Green L.C., Wagner D.A., Glogowski J., Skipper P.L., Wishnok J.S. and Tannenbaum S.R. (1982) Analysis of nitrate, nitrite, and [15N] nitrate in biological fluids. Anal. Biochem. 126, 131–138 10.1016/0003-2697(82)90118-X 7181105

[41] Akerboom T.P. and Sies H. (1981) Assay of glutathione, glutathione disulfide, and glutathione mixed disulfides in biological samples. Methods in enzymology, pp. 373–382, Elsevier 10.1016/s0076-6879(81)77050-2 7329314

[42] Aebi H. (1984) Catalase in vitro. Methods Enzymol. 105, 121–126 10.1016/S0076-6879(84)05016-3 6727660

[43] Misra H.P. and Fridovich I. (1972) The role of superoxide anion in the autoxidation of epinephrine and a simple assay for superoxide dismutase. J. Biol. Chem. 247, 3170–3175 10.1016/S0021-9258(19)45228-9 4623845

[44] Pinto M.C., Mata A.M. and Lopez-barea J. (1984) Reversible inactivation of Saccharomyces cerevisiae glutathione reductase under reducing conditions. Arch. Biochem. Biophys. 228, 1–12 10.1016/0003-9861(84)90040-7 6364985

[45] Tappel A. (1978) Glutathione peroxidase and hydroperoxides. Methods in enzymology, pp. 506–513, Elsevier 10.1016/s0076-6879(78)52055-7 672654

[46] Abdeen A., Samir A., Elkomy A., Aboubaker M., Habotta O.A., Gaber A. et al. (2021) The potential antioxidant bioactivity of date palm fruit against gentamicin-mediated hepato-renal injury in male albino rats. Biomed. Pharmacother. 143, 112154 10.1016/j.biopha.2021.112154 34649332

[47] Kassab R.B., Theyab A., Al-Ghamdy A.O., Algahtani M., Mufti A.H., Alsharif K.F. et al. (2022) Protocatechuic acid abrogates oxidative insults, inflammation, and apoptosis in liver and kidney associated with monosodium glutamate intoxication in rats. Environment. Sci. Pollution Res. 29, 12208–12221 10.1007/s11356-021-16578-4 34562213

[48] AL‐Megrin W.A., Metwally D.M., Habotta O.A., Amin H.K., Abdel Moneim A.E. and El‐khadragy M. (2020) Nephroprotective effects of chlorogenic acid against sodium arsenite‐induced oxidative stress, inflammation, and apoptosis. J. Sci. Food Agric. 100, 5162–5170 10.1002/jsfa.10565 32519758

[49] Oh S.-M., Park G., Lee S.H., Seo C.-S., Shin H.-K. and Oh D.-S. (2017) Assessing the recovery from prerenal and renal acute kidney injury after treatment with single herbal medicine via activity of the biomarkers HMGB1, NGAL and KIM-1 in kidney proximal tubular cells treated by cisplatin with different doses and exposure times. BMC Complement. Altern. Med. 17, 1–9 29258482 10.1186/s12906-017-2055-y PMC5738030

[50] Deng J.-S., Jiang W.-P., Chen C.-C., Lee L.-Y., Li P.-Y., Huang W.-C. et al. (2020) Cordyceps cicadae mycelia ameliorate cisplatin-induced acute kidney injury by suppressing the TLR4/NF-κB/MAPK and activating the HO-1/Nrf2 and Sirt-1/AMPK pathways in mice. Oxidative Med. Cell. Longevity 2020, 7912763 10.1155/2020/7912763 32089779 PMC7026739

[51] Abdelsalam M., Elmorsy E., Abdelwahab H., Algohary O., Naguib M., El Wahab A.A. et al. (2018) Urinary biomarkers for early detection of platinum based drugs induced nephrotoxicity. BMC Nephrol. 19, 1–8 10.1186/s12882-018-1022-2 30180818 PMC6123931

[52] Mosa I.F., Youssef M., Kamel M., Mosa O.F. and Helmy Y. (2019) Synergistic antioxidant capacity of CsNPs and CurNPs against cytotoxicity, genotoxicity and pro-inflammatory mediators induced by hydroxyapatite nanoparticles in male rats. Toxicol. Res. 8, 939–952 10.1039/c9tx00221a PMC7069401 32206303

[53] Anwar M., Muhammad F., Akhtar B. and Saleemi M.K. (2020) Nephroprotective effects of curcumin loaded chitosan nanoparticles in cypermethrin induced renal toxicity in rabbits. Environ. Sci. Pollut. Res. 27, 14771–14779 10.1007/s11356-020-08051-5 32056099

[54] Sadek K.M., Lebda M.A., Abouzed T.K., Nasr S.M. and Shoukry M. (2017) Neuro-and nephrotoxicity of subchronic cadmium chloride exposure and the potential chemoprotective effects of selenium nanoparticles. Metab. Brain Dis. 32, 1659–1673 10.1007/s11011-017-0053-x 28660360

[55] Zahran W.E., Elsonbaty S.M. and Moawed F.S.M. (2017) Selenium nanoparticles with low-level ionizing radiation exposure ameliorate nicotine-induced inflammatory impairment in rat kidney. Environ. Sci. Pollut. Res. Int. 24, 19980–19989 10.1007/s11356-017-9558-4 28691127

[56] El-Khadragy M.F., Al-Megrin W.A., Alomar S., Alkhuriji A.F., Metwally D.M., Mahgoub S. et al. (2021) Chlorogenic acid abates male reproductive dysfunction in arsenic-exposed mice via attenuation of testicular oxido-inflammatory stress and apoptotic responses. Chem. Biol. Interact. 333, 109333 10.1016/j.cbi.2020.109333 33242462

[57] Lokman M.S., Althagafi H.A., Alharthi F., Habotta O.A., Hassan A.A., Elhefny M.A. et al. (2022) Protective effect of quercetin against 5-fluorouracil-induced cardiac impairments through activating Nrf2 and inhibiting NF-κB and caspase-3 activities. Environ. Sci. Poll. Res. 30, 17657–17669 10.1007/s11356-022-23314-z 36197616

[58] Jia L., Xue K., Liu J., Habotta O.A., Hu L. and Moneim A.E.A. (2020) Anticolitic effect of berberine in rat experimental model: impact of PGE2/p38 MAPK pathways. Mediators Inflamm. 2020, 10.1155/2020/9419085 PMC7542536 33061833

[59] Pakfetrat M., Akmali M., Malekmakan L., Dabaghimanesh M. and Khorsand M. (2015) Role of turmeric in oxidative modulation in end-stage renal disease patients. Hemodialysis Int. Int. Symposium Home Hemodialysis 19, 124–131 10.1111/hdi.12204 25131305

[60] Edrees N.E., Galal A.A.A., Abdel Monaem A.R., Beheiry R.R. and Metwally M.M.M. (2018) Curcumin alleviates colistin-induced nephrotoxicity and neurotoxicity in rats via attenuation of oxidative stress, inflammation and apoptosis. Chem. Biol. Interact. 294, 56–64 10.1016/j.cbi.2018.08.012 30138604

[61] Ishaq A., Gulzar H., Hassan A., Kamran M., Riaz M., Parveen A. et al. (2021) Ameliorative mechanisms of turmeric-extracted curcumin on arsenic (As)-induced biochemical alterations, oxidative damage, and impaired organ functions in rats. Environ. Sci. Pollut. Res. Int. 28, 66313–66326 10.1007/s11356-021-15695-4 34331650

[62] Di Tu Q., Jin J., Hu X., Ren Y., Zhao L. and He Q. (2020) Curcumin Improves the Renal Autophagy in Rat Experimental Membranous Nephropathy via Regulating the PI3K/AKT/mTOR and Nrf2/HO-1 Signaling Pathways. BioMed. Res. Int. 2020, 7069052 10.1155/2020/7069052 33204708 PMC7654212

[63] Mosa I.F., Yousef M.I., Kamel M., Mosa O.F. and Helmy Y. (2019) The protective role of CsNPs and CurNPs against DNA damage, oxidative stress, and histopathological and immunohistochemical alterations induced by hydroxyapatite nanoparticles in male rat kidney. Toxicol. Res. 8, 741–753 10.1039/c9tx00138g PMC6764468 31588351

[64] AlBasher G., Alfarraj S., Alarifi S., Alkhtani S., Almeer R., Alsultan N. et al. (2020) Nephroprotective role of selenium nanoparticles against glycerol-induced acute kidney injury in rats. Biol. Trace Elem. Res. 194, 444–454 10.1007/s12011-019-01793-5 31264127

[65] Habotta O.A., Wang X., Othman H., Aljali A.A., Gewaily M., Dawood M. et al. (2022) Selenium-enriched yeast modulates the metal bioaccumulation, oxidant status, and inflammation in copper-stressed broiler chickens. Front. Pharmacol. 13, 1026199 10.3389/fphar.2022.1026199 36313334 PMC9614105

[66] Alsharif K.F., Albrakati A., Al Omairi N.E., Almalki A.S., Alsanie W.F., Elmageed Z.Y.A. et al. (2022) Therapeutic antischizophrenic activity of prodigiosin and selenium co-supplementation against amphetamine hydrochloride-induced behavioural changes and oxidative, inflammatory, and apoptotic challenges in rats. Environ. Sci. Pollut. Res. Int. 30, 7987–8001 10.1007/s11356-022-22409-x 36048389

[67] Xu G., Gu Y., Yan N., Li Y., Sun L. and Li B. (2021) Curcumin functions as an anti‐inflammatory and antioxidant agent on arsenic‐induced hepatic and kidney injury by inhibiting MAPKs/NF‐κB and activating Nrf2 pathways. Environ. Toxicol. 36, 2161–2173 10.1002/tox.23330 34272803

[68] Liu Z., Shi B., Wang Y., Xu Q., Gao H., Ma J. et al. (2022) Curcumin alleviates aristolochic acid nephropathy based on SIRT1/Nrf2/HO-1 signaling pathway. Toxicology 479, 153297 10.1016/j.tox.2022.153297 36037877

[69] Qiao L., Dou X., Yan S., Zhang B. and Xu C. (2020) Biogenic selenium nanoparticles synthesized by Lactobacillus casei ATCC 393 alleviate diquat-induced intestinal barrier dysfunction in C57BL/6 mice through their antioxidant activity. Food Funct. 11, 3020–3031 10.1039/D0FO00132E 32243488

[70] Kim J.-Y., Jo J., Kim K., An H.-J., Gwon M.-G., Gu H. et al. (2019) Pharmacological activation of Sirt1 ameliorates cisplatin-induced acute kidney injury by suppressing apoptosis, oxidative stress, and inflammation in mice. Antioxidants 8, 322 10.3390/antiox8080322 31431003 PMC6720310

[71] Othman M.S., Al-Bagawi A.H., Obeidat S.T., Fareid M.A., Habotta O.A. and Moneim A.E.A. (2022) Antitumor activity of zinc nanoparticles synthesized with berberine on human epithelial colorectal adenocarcinoma (Caco-2) cells through acting on Cox-2/NF-kB and p53 pathways. Anti-Cancer Agents Med. Chem. 22, 2002–2010 10.2174/1871520621666211004115839 34607550

[72] Li F., Xu Y., Li X., Wang X., Yang Z., Li W. et al. (2021) Triblock copolymer nanomicelles loaded with curcumin attenuates inflammation via inhibiting the NF-Κb pathway in the rat model of cerebral ischemia. Int. J. Nanomed. 16, 3173 10.2147/IJN.S300379 PMC8121676 34007172

[73] Zaghloul R.A., Abdelghany A.M. and Samra Y.A. (2022) Rutin and selenium nanoparticles protected against STZ-induced diabetic nephropathy in rats through downregulating Jak-2/Stat3 pathway and upregulating Nrf-2/HO-1 pathway. Eur. J. Pharmacol. 933, 175289 10.1016/j.ejphar.2022.175289 36122758

[74] Gu H., Gwon M.-G., Kim J.H., Leem J. and Lee S.-J. (2022) Oridonin attenuates cisplatin-induced acute kidney injury via inhibiting oxidative stress, apoptosis, and inflammation in mice. BioMed Res. Int. 2022, 3002962 10.1155/2022/3002962 35469348 PMC9034941

[75] Ahmed Z.S.O., Galal M.K., Drweesh E.A., Abou-El-Sherbini K.S., Elzahany E.A.M., Elnagar M.M. et al. (2021) Protective effect of starch-stabilized selenium nanoparticles against melamine-induced hepato-renal toxicity in male albino rats. Int. J. Biol. Macromol. 191, 792–802 10.1016/j.ijbiomac.2021.09.156 34597692

