# Correction: Therapeutic activity of green synthesized selenium nanoparticles from turmeric against cisplatin-induced oxido-inflammatory stress, and cell death in mice kidney

**DOI:** 10.1042/BSR-2023-1130_COR

**Published:** 2024-10-29

**Keywords:** cisplatin, inflammation, selenium nanoparticles, turmeric extract

The authors of the original article “Therapeutic activity of green synthesized selenium nanoparticles from turmeric against cisplatin-induced oxido-inflammatory stress and cell death in mice kidney” (10.1042/BSR20231130) would like to correct the affiliations of the author Bassel Dawod.

The author was originally listed with the following affiliations:

- McMaster Children's Hospital, Faculty of Health Sciences, Hamilton, Ontario, Canada
- Department of Biology, College of Science, Jouf University, Sakaka, Al-Jouf, Saudi Arabia
- Department of Zoology, Faculty of Science, Fayoum University, Fayoum, Egypt

The correct affiliation is McMaster Children's Hospital, Faculty of Health Sciences, Hamilton, Ontario, Canada. The two additional affiliations have now been removed for this author.

The authors would also like to correct the funding statement which was originally given as:

“This work was funded by the Deanship of Scientific Research at Jouf University under grant No (DSR-2021-03-03112).”

This should instead state:

“This work was funded by the Deputyship for Research & Innovation, Ministry of Education in Saudi Arabia for funding this research work through the project number 223202.”

